# The Regulated Secretory Pathway in Neuroendocrine Cells

**DOI:** 10.3389/fendo.2014.00048

**Published:** 2014-04-08

**Authors:** Rafael Vazquez-Martinez, Stéphane Gasman

**Affiliations:** ^1^Department of Cell Biology, Physiology and Immunology, Instituto Maimónides de Investigaciones Biomédicas de Córdoba (IMIBIC), Reina Sofia University Hospital, University of Córdoba, Córdoba, Spain; ^2^CIBER Fisiopatología de la Obesidad y Nutrición (CIBERobn), Córdoba, Spain; ^3^Centre National de la Recherche Scientifique (CNRS UPR 3212), Institut des Neurosciences Cellulaires et Intégratives (INCI), Université de Strasbourg, Strasbourg, France

**Keywords:** secretion, neuroendocrine cells, membrane trafficking, large dense core vesicles, regulated exocytosis, endocytosis, super-resolution microscopy

The regulated secretory pathway shared by excitable cells, including neurons and neuroendocrine cells is an intricate process that comprises multiple, tightly regulated steps. After their synthesis in the endoplasmic reticulum, hormones and neuropeptides have to be sorted and packed into large dense core vesicles (also named secretory granules) in the Golgi apparatus. Granules are transported toward the plasma membrane in a cytoskeleton-dependent manner and mature into competent organelles for secretagogue-induced exocytosis. Granules are then tethered to the plasma membrane, docked, and primed, before finally releasing their contents after fusing with the plasma membrane. To ensure that neurotransmission and neuroendocrine secretion operate correctly, all these steps must be tightly regulated and coordinated both spatially and temporally. Currently, when the field of intracellular trafficking has been honored by the 2013 Nobel Prize in Physiology or Medicine (awarded to James Rothman, Randy Schekman, and Thomas Südhof for their pioneering works on vesicular transport), this issue of Frontiers in Neuroendocrine Science is aimed to providing an up-to-date overview of the cellular and molecular mechanisms governing the regulated secretory pathway in neuroendocrine cells. Reviews presented here are widely covering this topic, from the architecture of the organelle involved in secretory cargo processing and sorting, the biogenesis of secretory granules, their specific transport toward the plasma membrane to the late steps of exocytosis, and the secretory granule membrane recapture.

Early stages of the secretory pathway have been discussed by two groups. Emma Martinez-Alonso and colleagues discuss the Golgi complex architecture, as well as the regulatory proteins that govern extra- and intra-Golgi transport, and the still controversial, but not mutually exclusive, theoretical models proposed to explain cargo progression through the Golgi stack (1). The group of Richard Mains discusses the specific roles of cytosolic adaptor proteins such as AP-1A, PACS-1, and GGAs in the assembly and maturation of secretory granules (2).

Closer to the cell surface, several groups discuss the molecular mechanisms regulating the late stage of exocytosis. The group of Frédéric Meunier reviews recent insights of the role of the cortical acto-myosin network (3), whereas the role of different tethering and priming factors such as CAPS, Munc13, and Doc2 proteins is described by the groups of Ury Ashery and Tom Martin (4, 5). Paanteha Moghadam and Meyer Jackson review how various synaptotagmins regulate fusion pore kinetics and control the mode of release (6). Lipids have emerged as key players of the regulated exocytosis and the group of Nicolas Vitale presents an overview on the diverse roles that lipids play in defining exocytotic sites, both by affecting membrane topology and by regulating secretory vesicle priming and fusion (7).

Finally, exocytosis cannot exist without a compensatory membrane intake process (i.e., endocytosis), which allows recycling of granule components and maintains organelle integrity. The groups of Stéphane Gasman and Alla Rynditch discuss the mechanisms that coordinate clathrin-mediated compensatory endocytosis with exocytosis, highlighting the specific role of the intersectin family of scaffold proteins in exocytosis and endocytosis (8, 9). The group of Ana-Maria Cardenas reviews the pleiotropic role of the mechano-GTPase dynamin-2, on intracellular membrane fission and fusion events, vesicle traffic, and cytoskeleton dynamics, as well as the impact of dynamin-2 mutations on the correct functioning of the secretory pathway (10).

On a more physiological point of view, Maria-Luisa Durán-Pasten and Tatiana Fiordelisio present an example of how pituitary gonadotrophs receive and transduce extracellular signals to promote luteinizing (LH) and follicle-stimulating (FSH) secretion, highlighting the tremendous plasticity of the system for adapting to different physiological demands (11). Wei-Jye Lin and Stephen Salton report that single nucleotide polymorphisms in genes encoding secreted proteins are associated with neuropsychiatric or endocrine/metabolic disorders (12). Finally, Jennifer Fitch-Tewfik and Robert Flaumenhaft demonstrate how the regulated secretory pathway is similar in mast cells compared to neuroendocrine cells from the adrenal gland (13), and Burton Dickey’s group describes the regulatory mechanism of mucin secretion in a non-neuroendocrine cell model (14).

On a more technical point of view, recent improvements in detection technologies, especially in optical microscopy, continually push the limits of sensitivity and resolution. The groups of Colin Rickman and Ute Becherer discuss how advances over the last decade in fluorescence microscopy provided spatial and temporal details on the subcellular organization of the molecular machinery governing the regulated secretory pathway (15, 16), whereas the group of Rory Duncan describes how the combination of new imaging approaches with super-resolution microscopy and novel calcium indicators is appropriate for accurate study of voltage-gated calcium channel locations, interactions, dynamics, and composition in living cells (17).

Collectively, this compilation of reviews intends to illustrate the recent progress made to understand the complex regulation of the granule secretory pathways in neuroendocrine cells. We are grateful to all the authors who have contributed to this Research Topic and to the dedicated reviewers who helped us reaching the highest quality standards.

## Conflict of Interest Statement

The authors declare that the research was conducted in the absence of any commercial or financial relationships that could be construed as a potential conflict of interest.
